# Two modes of fusogenic action for influenza virus fusion peptide

**DOI:** 10.1371/journal.pcbi.1011174

**Published:** 2023-05-26

**Authors:** Michal Michalski, Piotr Setny

**Affiliations:** Centre of New Technologies, University of Warsaw, Warsaw, Poland; Heidelberg Institute for Theoretical Studies (HITS gGmbH), GERMANY

## Abstract

The entry of influenza virus into the host cell requires fusion of its lipid envelope with the host membrane. It is catalysed by viral hemagglutinin protein, whose fragments called fusion peptides become inserted into the target bilayer and initiate its merging with the viral membrane. Isolated fusion peptides are already capable of inducing lipid mixing between liposomes. Years of studies indicate that upon membrane binding they form bend helical structure whose degree of opening fluctuates between tightly closed hairpin and an extended boomerang. The actual way in which they initiate fusion remains elusive. In this work we employ atomistic simulations of wild type and fusion inactive W14A mutant of influenza fusion peptides confined between two closely apposed lipid bilayers. We characterise peptide induced membrane perturbation and determine the potential of mean force for the formation of the first fusion intermediate, an interbilayer lipid bridge called stalk. Our results demonstrate two routes through which the peptides can lower free energy barrier towards fusion. The first one assumes peptides capability to adopt transmembrane configuration which subsequently promotes the creation of a stalk-hole complex. The second involves surface bound peptide configuration and proceeds owing to its ability to stabilise stalk by fitting into the region of extreme negative membrane curvature resulting from its formation. In both cases, the active peptide conformation corresponds to tight helical hairpin, whereas extended boomerang geometry appears to be unable to provide favourable thermodynamic effect. The latter observation offers plausible explanation for long known inactivity of boomerang-stabilising W14A mutation.

## Author summary

In order to deliver genetic material into host cells enveloped viruses, such as Influenza, HIV or Corona, need to fuse their lipid envelope with the cell membrane. This process is mediated by viral fusion proteins, whose specialised elements, called fusion peptides, are inserted into target membranes and initiate their merging with the viral lipid bilayer. Being dynamic and dependent on individual molecules, this process is inherently difficult to study, and hence, its details remain elusive. Here, we report results of fully atomistic simulations aimed at revealing the mode of action of influenza virus fusion peptides. Aside from analysing structural aspects of peptide-induced lipids perturbation, we calculate free energy changes during the formation of the first fusion intermediate between two closely apposed lipid bilayers, that is a hydrophobic bridge called stalk. We demonstrate that the peptides are indeed capable of lowering the associated free energy barrier in comparison to membrane only system. They appear to do so via two possible routes. They can either remain at the membrane-water interface and stabilise stalk by shielding its core from aqueous environment, or they can adopt membrane-spanning configurations and lower the cost of membrane deformation by forming so called stalk-hole complexes.

## Introduction

Membrane fusion is a central event during entry of enveloped viruses into host cells [1]. In the case of influenza virus it involves merging of its lipid envelope with the membrane of an endosomal vesicle, in which the virus is encapsulated upon internalisation [2]. The process is catalysed by viral surface homotrimeric protein, hemagglutinin (HA) [3, 4]. Upon its cleavage by host proteases into HA1 and HA2 subunits and activation by acidic endosomal environment, HA2 folds into an elongated coiled-coil structure directed outwards from the virus surface [4, 5]. It enables insertion of N-terminal HA2 fragments, called fusion peptides (HAfp), into the target membrane providing an anchor point for its subsequent drawing to the viral envelope [6–8]. Overcoming the related dehydration barrier is deemed possible owing to partial HA2 refolding during which its sections located within the C-terminal half pack along the coiled-coil stem [9].

**Fig 1:**
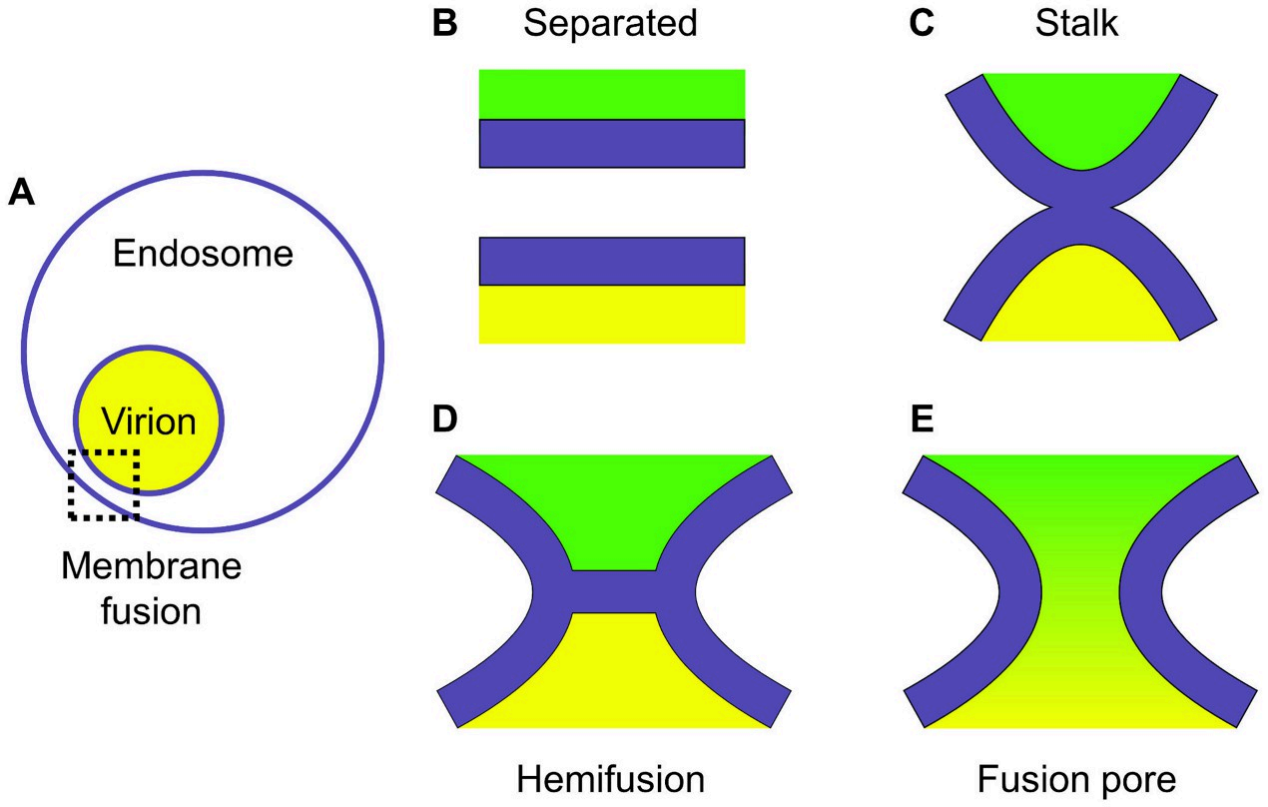
Schematics of membrane fusion stages. The membranes are shown in blue, cell and virion environments in green and yellow, respectively. (A) Tight contact of viral and endosomal membranes. (B) Two apposed lipid bilayers. (C) Stalk, a hydrophobic lipid bridge between proximal monolayers. (D) Hemifusion diaphragm: during stalk expansion, lipids of the distal monolayers are brought into contact, thus forming a bilayer. (E) Fusion pore.

The actual fusion begins once the two membranes become closely apposed. It is believed that the dominant pathway involves nucleation of a hydrophobic lipid bridge between proximal monolayers, so called stalk, its transition into hemifusion diaphragm, and finally, the formation and expansion of an aqueous channel connecting the interiors of membrane enclosed structures (Fig 1) [10, 11]. This classic model, which implies permanent continuity of lipid envelope around the interacting bodies, is challenged, however, by the observation of possible content leakage during HA-mediated fusion [12, 13]. Indeed, a mechanism involving temporary rupture of the target membrane which precedes the formation of hemifusion diaphragm was directly observed by cryoelectron microscopy suggesting the existence of an alternative pathway [12]. An increased likelihood of such a pathway was associated with low cholesterol content within the target bilayer [12], which was later interpreted more generally in terms of membrane composition favouring spontaneous negative curvature [13].

A critical role in the early stages of fusion is played by HAfps [14]. They apparently act not merely as passive anchors aiding in the apposition of two membranes by HA2 subunits, but specifically contribute to the formation of lipid bridge between them. The sequence of 23 N-terminal HA2 amino acids which establish direct contact with the target membrane shows the highest level of conservation within the entire HA protein [15]. A number of point mutations in this region are known to completely abrogate fusion or arrest it at an intermediate state, indicating high level of specialisation [16]. Notably, synthetic HAfps, are already capable of inducing lipid mixing between liposomes without the aid of the rest of the fusion machinery [17]. Similar dependence of their activity on amino acids substitutions or pH variations to the one observed for complete HA protein indicates that they provide all functionality necessary for early fusion events, serving as a good model for its studies [18].

HAfps are unfolded in aqueous solution, but once in the membrane they adopt alpha helical structure with a kink centred at residue 13 [19–21]. Most likely, their confuguration fluctuates between more or less open boomerang-like geometry and closed helical hairpin [22, 23]. A conservation of a number of glycine residues in arrangement permitting the formation of a sharp kink and tight antiparallel packing of two helices, as well as the effect of W14A mutation which inhibits fusion while promoting overly flexible boomerang structure [24], suggest that the hairpin may be the actual fusion competent geometry. The detailed knowledge concerning HAfp location and orientation within lipid bilayer is still missing. According to the most popular view they remain at the lipid-water interface [21, 25] or are shallowly inserted into the proximal membrane leaflet with solvent-facing kink region [20, 26–28]. There is also, however, experimental evidence of deep intramembrane HAfp location [29]. This variability is supported by atomistic molecular dynamics (MD) simulations [30–34], which in addition point to the possibility of fully transmembrane (TM) configuration whose stability is maintained owing to membrane indentation which provides contact with aqueous environment for both hairpin poles [33, 35–37]. Recent simulations of multiple, membrane bound HAfp units demonstrate their ability to aggregate and displace lipids from the cis leaflet leading to overall membrane thinning and increased susceptibility to poration [38].

The apparent abundance of different outcomes stems from possibly natively diverse HAfp configuration space and its sensitivity to subtle effects such as pH or temperature, but also from the variety of considered lipid compositions and spatial organisations. Additional complication arises from the fact that membrane fusion is a dynamic event which relies on individual peptide and lipid molecules but at the same time on mesoscopic properties of membrane and aqueous compartment. Given the above, the actual mechanism of HAfp fusogenic activity remains elusive. To this end, multiple options have been proposed: local dehydration of intermembrane space [39], stabilisation of positive membrane curvature [40], stabilisation of negative membrane curvature [25], membrane thinning [41, 42], membrane rupture by TM bundles of boomerang-like HAfps [43], lipid heads intrusion [31], and promotion of lipid tails protrusions [30]. This latter mechanism is particularly appealing since, according to general view on early fusion events, splaying of lipid acyl chains is the first step leading to stalk formation [44, 45]. Along those lines, HAfp-induced lipid tails protrusions have been indeed reported to underlie lipid mixing in a recent multi scale simulation of full-length HA mediated fusion of lipid vesicle with planar membrane [46]. On the other hand, however, the difference in the frequency of lipid tail protrusions around surface bound wild type (wt) HAfp and fusion incompetent mutants observed in other MD studies [30, 31, 36] seems to be too small (less than twofold) to convincingly explain inactivity of the latter.

Detailed, quantitative investigation of fusion in the presence of protein components requires system representation in atomistic or near atomistic resolution, which significantly limits the applicability of theoretical models based on elasticity theory [47]. Simulation based studies focussing on systems of already apposed lipid membranes [44, 48–50] indicate free energy barrier for stalk formation of at least 30 kJmol^−1^ in low hydration regime of intermembrane space, with a significant tendency to grow along with increasing bilayers separation, *d*. Stalk structures that are energetically favourable with respect to two planar membranes are found for *d* ≲ 0.9 nm, while already above *d* ∼ 1.3 nm their associated free energy minimum vanishes, indicating inherent lack of stability [49]. Although all studies consistently observe splayed lipid tails as an important intermediate state on the route to stalk formation, the appearance of splayed configurations that were truly related to fusion was observed to involve at least 75% of the entire free energy barrier [44]. Accordingly, it is not clear whether they can be identified with protrusions of lipid tails whose promotion by surface bound HAfps was observed in unperturbed MD runs discussed above [30, 31, 36].

The fusogenic effect of a protein was investigated directly for the transmebrane SNARE domain [49]. It manifested itself by stalk stabilisation and the reduction of free energy barrier for its formation, which were attributed to effective softening of the membrane and lowering excess free energy of highly bent stalk structure [49], rather than to perturbation of lipid tails. Whereas individual protein systems most likely differ in details of their fusogenic activity, this result illustrates that capturing the actual mechanism requires detailed modelling of protein, lipid and aqueous components throughout the entire process of stalk formation.

In the current work we use fully atomistic MD simulations to investigate the role of HAfp in the formation of an initial lipid bridge between two closely apposed bilayers. First, using unconstrained MD we assess peptides influence on the interbilayer space looking for perturbation that would be compatible with the expected fusogenic activity. Second, we carry out umbrella sampling simulations to calculate the potential of mean force (PMF) for stalk formation in peptides presence at two different temperatures. The estimated thermodynamic parameters are compared with results obtained for analogous membrane only systems in order to pinpoint peptide-related effects.

## Results and discussion

### Unconstrained pre-stalk systems

**Fig 2:**
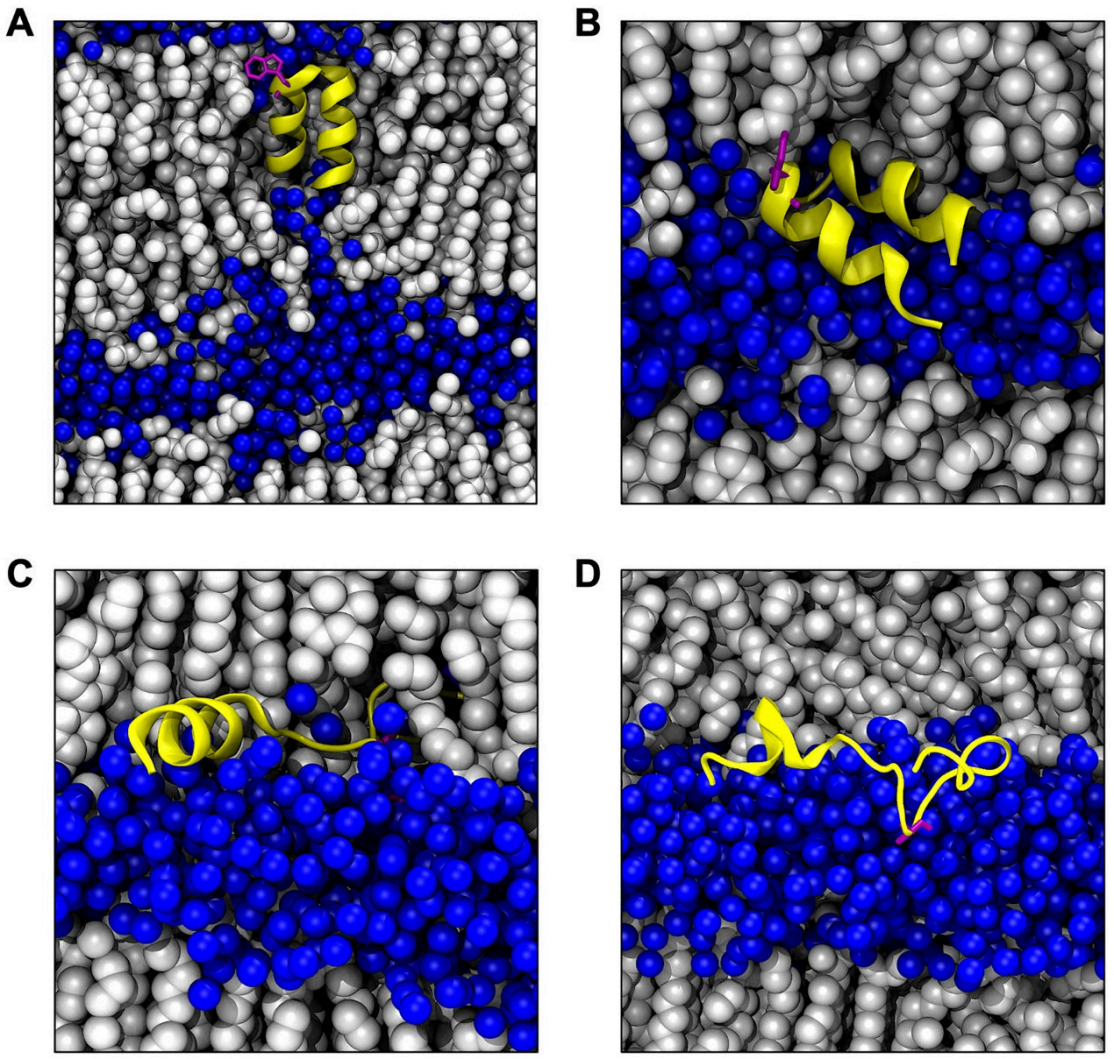
Simulation snapshots of considered HAfp configurations: (A) TM hairpin, (B) surface hairpin, (C) boomerang, (D) W14 mutant. Color coding: water oxygen atoms, blue spheres; lipid and cholesterol molecules: white spheres, HAfp: yellow ribbon; HAfp Trp14 side chain: purple.

In order to investigate the effect of peptides presence between two closely apposed bilayers, we performed unconstrained molecular dynamics simulations of systems consisting of two lipid membranes separated by *d* ∼ 1 nm layer of aqueous solvent, with a single HAfp confined in the intermembrane space. We considered four different scenarios: 1) surface bound wt HAfp in hairpin or 2) boomerang conformation, 3) surface bound inactive W14A mutant in extended configuration, and 4) TM wt hairpin (Fig 2). Each variant was subjected to two, 500+ ns, independent atomistic MD runs, of which the final 400 ns were combined for analysis. For comparison, a membranes-only system simulated in analogous conditions was also considered (see S1 Text for a detailed list of simulations).

**Fig 3:**
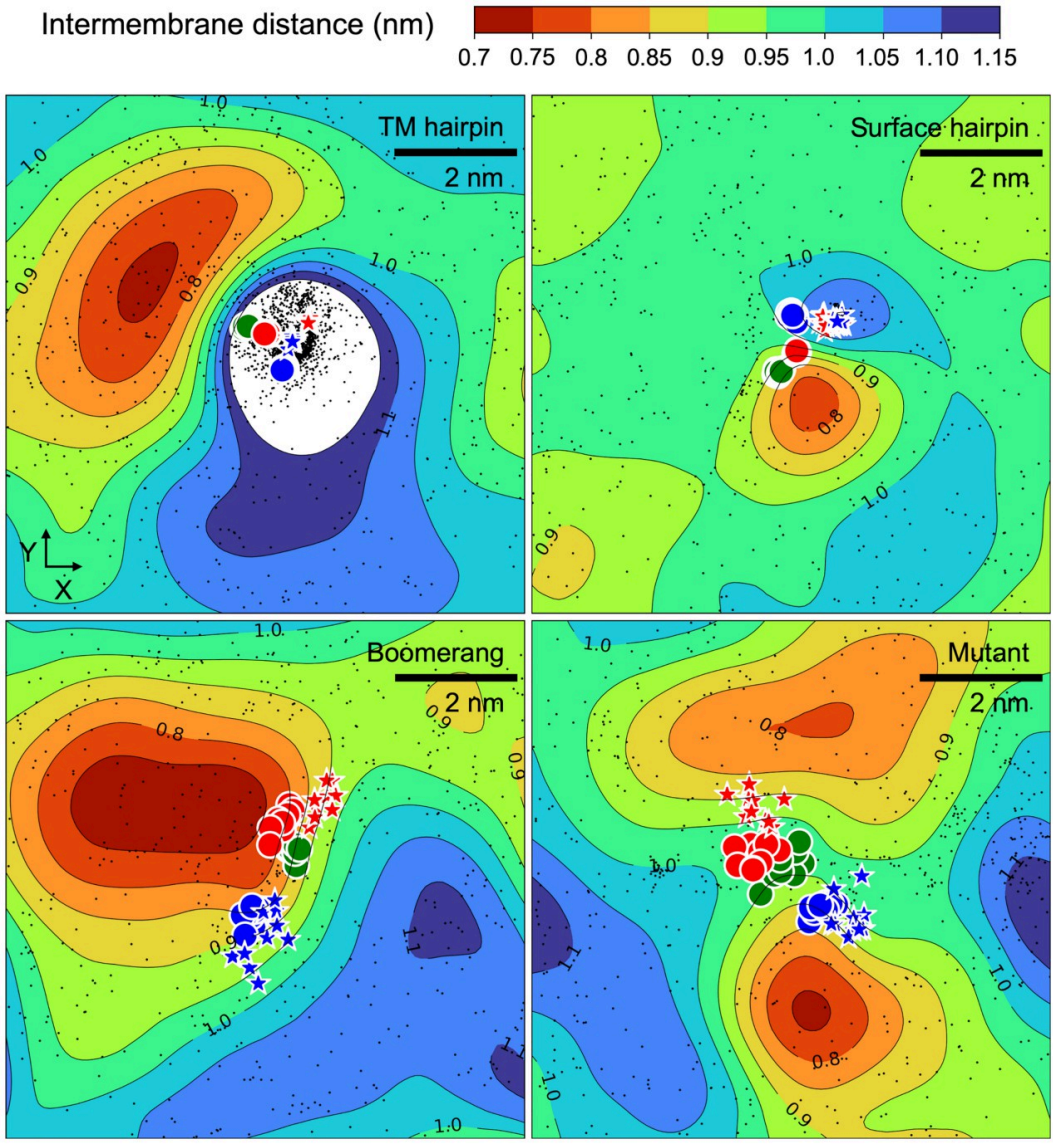
Thickness of interbilayer space averaged over 2 × 400 ns from independent MD runs. Positions of HAfp C*α* atoms every 100 ns are marked with circles; blue: residues 1, 7, 9, green: residue 14, red: residues 17, 21, 23; positions of N and C termini are marked by blue and red stars, respectively. Black dots correspond to locations of lipid tails protrusions.

**Fig 4:**
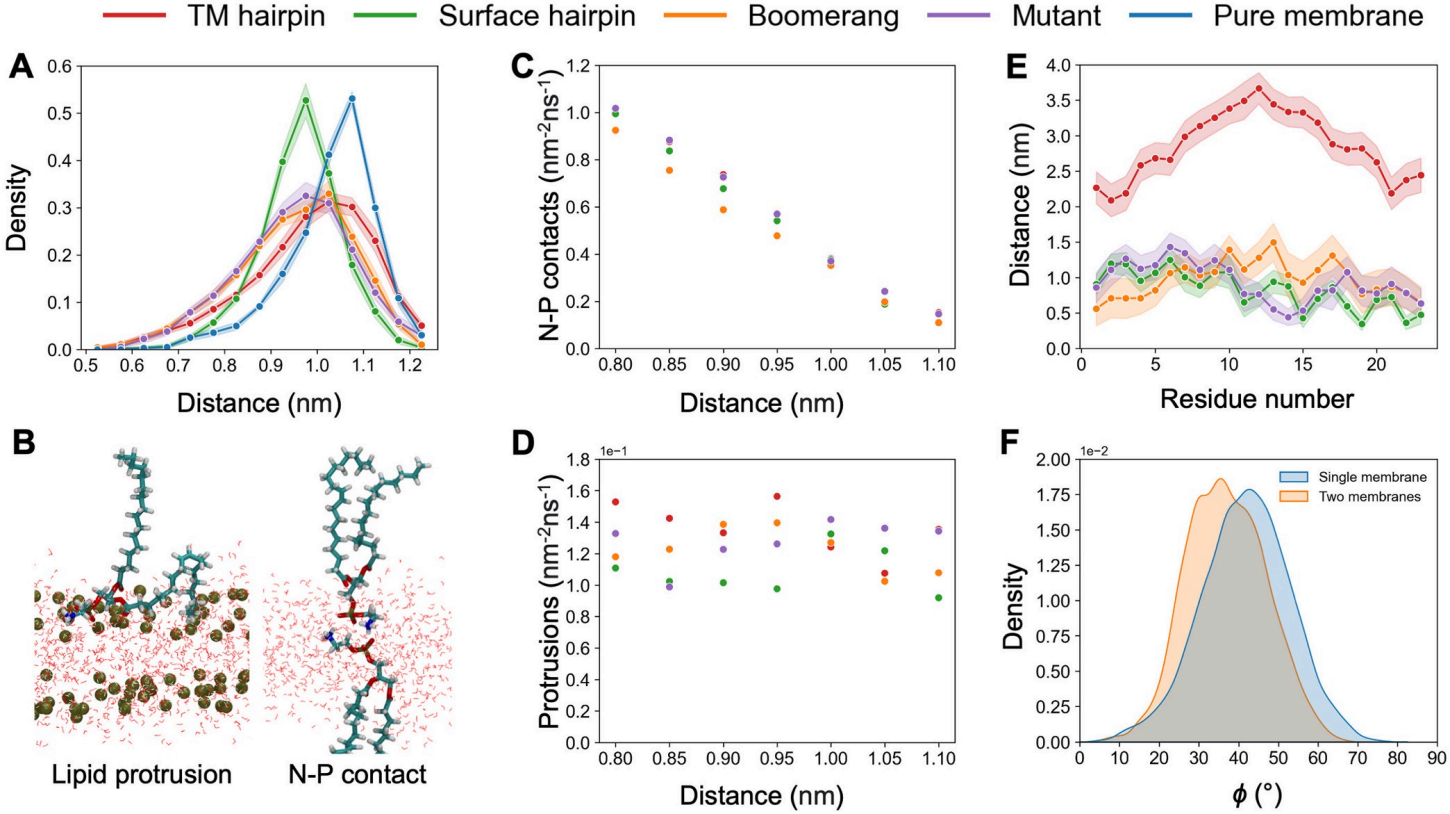
(A) Distributions of local (0.5 nm × 0.5 nm bins in *XY* plane) instantaneous interbilayer distances in considered systems. (B) Sample geometries of lipid tail protrusion and N-P contact between lipids from the opposing membranes. (C-D) Correlation between local interbilayer distance and the frequency of N-P contacts (C) or lipid tails protrusions (D). (E) Residue-based mean minimum distance profiles between HAfp and opposing membrane heavy atoms. (F) The distribution of *ϕ* angle (see text for definition) for surface HAfp hairpin in single and double bilayer systems. Shadows (A, E) correspond to one standard deviation.

In general, the peptides showed a tendency to promote dehydration of the interbilayer space in their vicinity (Fig 3). A more pronounced effect was found in the case of both boomerang structures and HAfp in TM configuration, while surface bound hairpin had relatively moderate impact. Notably, the observed average picture stems from modulation of spontaneous fluctuations of local interbilayer distances rather than stabilisation of static membrane deformation. The instantaneous thickness of the interbilayer space in our simulations was found to be highly uneven across time and space. Even in the membrane-only system, in which the interface was uniform in terms of molecular composition, the pattern of areas with diminished and increased separations fluctuated on a time scale of tens of ns (see Fig E in S1 Text). In all cases, the distribution of local interbilayer distances was clearly skewed towards lower values (Fig 4A), indicating a reduction in membranes repulsion once the thinning of water layer allowed direct interactions of polar lipid headgroups. The peptides apparently stabilised membrane contact patches in their vicinity and further enhanced the dehydration, which manifested itself by an increase in the fraction of the lowest interbilayer separations compared to the one observed in the membrane-only system (Fig 4A).

Assuming that the promotion of tight contact patches should facilitate lipid mixing, the amplitudes of the observed effects did not correlate with the expected fusogenic activity of surface-bound HAfp variants, of which the hairpin is deemed as the active one [21]. Its moderate impact on membrane separation may be attributed to the fact that it was found to lie flat at lipid-water interface, thus pointing its cluster of hydrophobic residues towards bilayer core and exposing relatively polar face [25] towards the opposing membrane. Such a stable arrangement was not maintained in the case of extended boomerang structures, and hence, their hydrophobic side chains were frequently present at the membrane surface, disrupting its polar character and promoting water depletion.

The degree of local dehydration was enough to warrant direct interactions (0.4 nm distance between terminal nitrogen and phosphorus atoms) between lipids from the opposing leaflets (Fig G in S1 Text). The underlying geometries involved protrusion and bending of lipid heads, resulting in the exposure of negatively charged phosphate groups for contact with positively charged terminal groups of lipids from the opposing membrane (Fig 4B). The frequency of such events was positively associated with the degree of intermembrane space thinning (Fig 4C). Somewhat surprisingly, however, no such correlation was observed for the frequency of acyl chains protrusions (Fig 4D), which are customary considered as an indicator of fusogenic activity [30]. The only evident increase in protrusions intensity could be observed in the vicinity of the TM HAfp hairpin (Fig 3), but this should be ascribed to deep local membrane indentation occurring in this system, rather than surface dehydration [36].

The peptides themselves did not show a tendency to insert into the opposing membrane within the considered simulation time. The most consistent interactions across the interbilayer space were maintained by solvent-exposed residues within the C-terminal helix of surface bound wt HAfp hairpin (Fig 4E). Nonetheless, the C-terminal helix did not seem to be specifically attracted towards the opposing leaflet, as can be judged based on the analysis of an angle, *ϕ*, formed between hairpin plane and membrane normal (*Z* axis of the system). A tendency to insert the C-terminal helix into the apposed membrane would shift hairpin orientation resulting in an increase in this angle compared to a situation, in which no second membrane is present. Instead, as evidenced by corresponding angle distributions shown in Fig 4F, a slight opposite effect was actually observed.

Taken together, these observations do not point to any obvious fusogenic effect of surface bound HAfps confined between already apposed membranes, that could be correlated with their expected activities. Possibly, peptide-dependent membrane perturbation that would promote stalk formation is a rare event, i.e. occurs at time scales or energy levels inaccessible in our unconstrained simulations, or requires cooperation of several peptide units. In this latter respect, surface bound wt HAfps seem to indeed adopt distinct position relative to the dehydrated region, giving more prospect for specific interactions than the W14A mutant. Both wt peptides consistently orient themselves such that their Trp14 residues remain anchored to the membrane contact patch (Fig 3). This orientation is particularly well maintained in the case of hairpin geometry, which in addition shows less conformational variability compared to the boomerang structure. The fusion-incompetent mutant, in which the Trp14 is exchanged to Ala, shows the greatest conformational disorder among all surface variants, and does not seem to be specifically attached to the dehydrated region.

### Stalk formation

**Fig 5:**
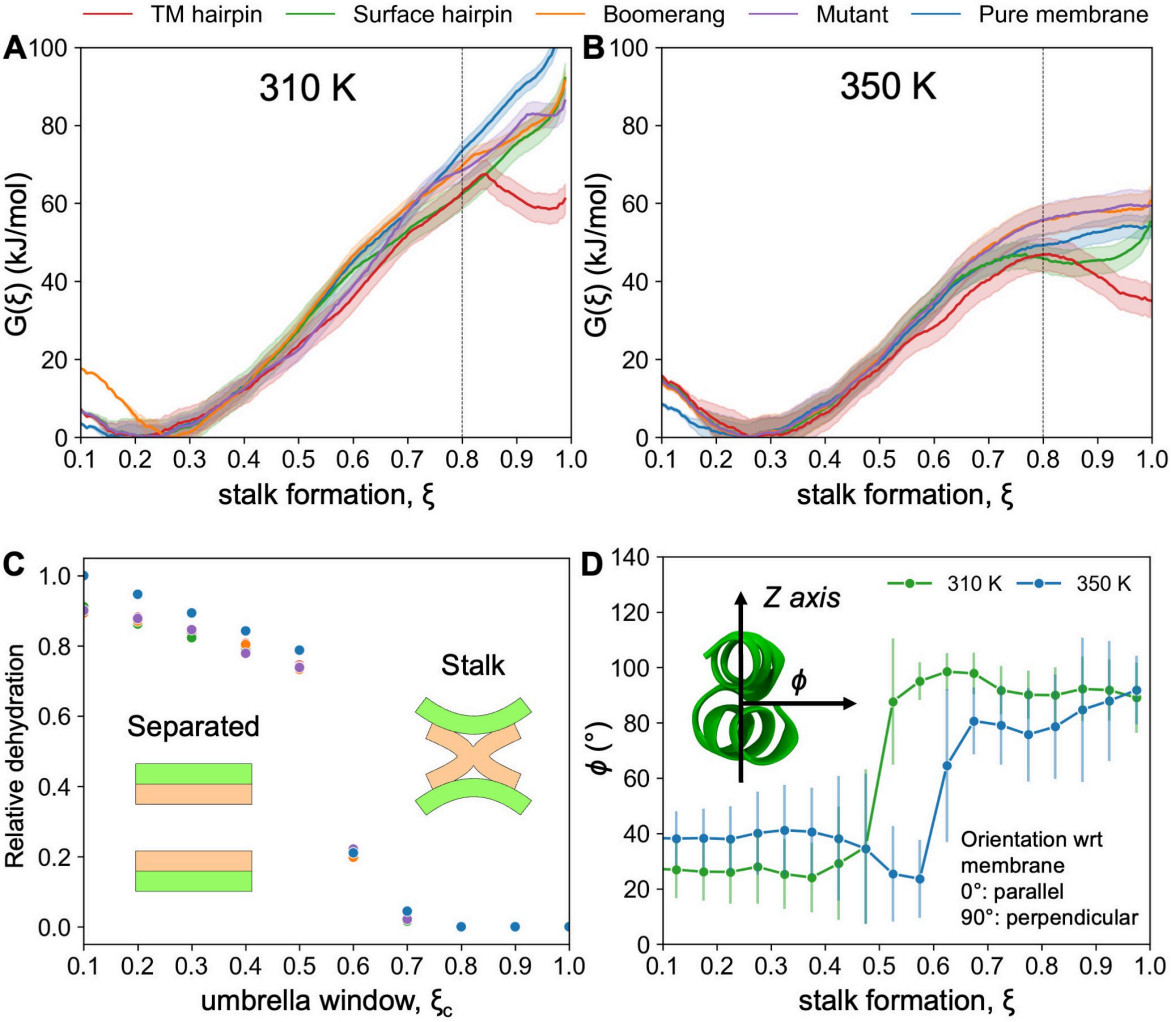
(A-B) PMF for stalk formation in (A) 310 K and (B) 350 K; vertical dashed lines indicate the moment of stalk formation assessed by the appearance of hydration discontinuity as shown in panel C. (C) Local dehydration of interbilayer space at 310 K at consecutive umbrella windows, relative to membrane-only system at *ξ*_*c*_ = 0.1 (see Methods for definition). (D) Mean angle (*ϕ*) between the orthogonal vector to surface-bound HAfp hairpin plane and the *Z* axis. Lines are guide to the eye, error bars correspond to one standard deviation.

In order to assess the thermodynamic effect of peptides presence on the formation of stalk and its subsequent stability, we calculated respective PMFs (Fig 5A and 5B) using reaction coordinate, *ξ*, proposed by Hub et al. [50]. In our implementation, *ξ* ∼ 0.2 corresponds to a system state with two parallel bilayers remaining at ∼ 1 nm distance, and *ξ* > 0.8 to states in which the membranes are connected by a stalk structure. The actual moment of stalk formation, around *ξ* = 0.8, is determined by the appearance of discontinuity in the aqueous layer separating the two membranes (Fig 5C). We assume that the work needed to reach this state starting from unperturbed, parallel membranes, is a free energy barrier, *G*^‡^, for stalk formation, regardless of whether the resulting stalk is stable or not. We note that the assumed moment of stalk formation corresponds to rather early stage of membranes contact, likely before passing the actual free energy barrier in case, in which a metastable stalk would truly form. Nonetheless, corresponding to an objective, system-independent criterion, the adopted definition serves well for the assessment of relative effects between the considered variants.

The PMF obtained at 310 K for membranes with no peptide, indicates *G*^‡^ in the order of 74 kJmol^−1^ (Table 1), and lacks minimum that would correspond to a stable stalk structure (Fig 5A). It is in qualitative agreement with coarse grained simulation results for analogous systems [44, 49, 50] conducted in relatively high hydration regime of the interbilayer space. According to the same studies, lowering water content is expected first to reduce the free energy barrier for stalk formation, and second, to stabilise the stalk structure. As the above effects were observed already for small changes in membrane separation, in the order of 0.01 nm [49, 50], we ensured strictly uniform average hydration of intermembrane space at the border of the simulation box in all considered systems (Fig A in S1 Text), such that to be able to focus exclusively on peptide-induced effects.

**Table 1:** Gibbs free energy (*G*^‡^, kJmol^−1^), entropy (ΔS, kJmol^−1^*K*^−1^), and enthalpy (ΔH, kJmol^−1^), corresponding to free energy barriers for stalk formation at 310 K and 350 K (in brackets). *T*Δ*S* is reported for 310 K.

Configuration	*G*^‡^ (kJmol^−1^)	ΔS (kJmol^−1^*K*^−1^)	TΔS (kJmol^−1^)	ΔH (kJmol^−1^)
Pure membrane	74 ± 2 (49 ± 3)	0.6 ± 0.1	186 ± 28	260 ± 28
Mutant	68 ± 3 (56 ± 4)	0.3 ± 0.1	93 ± 39	161 ± 39
Boomerang	68 ± 2 (56 ± 4)	0.3 ± 0.1	93 ± 35	161 ± 35
Surface hairpin	62 ± 4 (46 ± 4)	0.4 ± 0.1	124 ± 44	186 ± 44
TM hairpin	60 ± 4 (46 ± 5)	0.4 ± 0.2	124 ± 50	184 ± 50

The effect of peptides presence on the slope of PMF is not particularly evident in the lower range of the reaction coordinate (Fig 5A and 5B). This is in agreement with observations discussed above with respect to unconstrained simulations, in which there were no obvious peptide-induced perturbations that could be correlated with their expected activities. Around *ξ* = 0.8, however, all HAfps are shown to decrease the free energy barrier for stalk formation at 310 K in comparison to the one observed in peptide-free scenario. A greater shift in the barrier height originating from peptide presence, $\Delta G^{‡}=G_{pept}^{‡}-G_{nopept}^{‡}$, was observed for wt HAfps in hairpin conformation, reaching Δ*G*^‡^ ∼ −14 kJmol^−1^, in TM and Δ*G*^‡^ ∼ −12 kJmol^−1^ in surface configurations, whereas boomerang-like wt HAfp and W14A mutant led to Δ*G*^‡^ ∼ −6 kJmol^−1^.

At elevated temperature of 350 K, free energy barriers for stalk formation were considerably lower than at 310 K in all cases (Table 1). Assuming a crude estimate of entropy, $\Delta S=-(G_{350K}^{‡}-G_{310K}^{‡})/\Delta T$, with Δ*T* = 40 K, this indicates generally favourable entropic contribution and unfavourable enthalpy change during stalk formation, in qualitative agreement with experimental assessments [51]. Such thermodynamic signature can be expected to arise from disorganisation of lipid packing within the membrane core, and hydration defects caused by membrane surface perturbation accompanying stalk formation. Our qualitative estimates of thermodynamic contributions indicate that all peptides decrease the unfavourable enthalpy change at the expense of favourable entropic component. Particularly low magnitude of favourable entropic effect was observed in the case of boomerang structures. Accordingly, compared to the reference, peptide-free system at 350 K, only TM and surface-bound wt HAfp in hairpin conformation were observed to lower *G*^‡^, whereas both boomerang structures turned out to act against stalk formation.

We note that our calculations were performed based on simulations utilising moderately-sized system, modelled with the use of periodic boundary conditions. An increase in system size is expected to enhance fluctuations in lipid ordering and local curvature, possibly aiding in stalk formation. Likewise, larger membrane area is expected to allow more efficient accommodation of membrane strain within highly curved stalk region. Further lowering of stalk free energy might arise from convex membrane interface which likely forms at the fusion site owing to target membrane modelling by complete HA proteins. Accordingly, we believe that the obtained free energy barriers for stalk formation likely represent the upper limit for biologically relevant phenomena, at the same time preserving relative magnitudes of peptide induced contributions.

### Stalk structures and their stability

**Fig 6:**
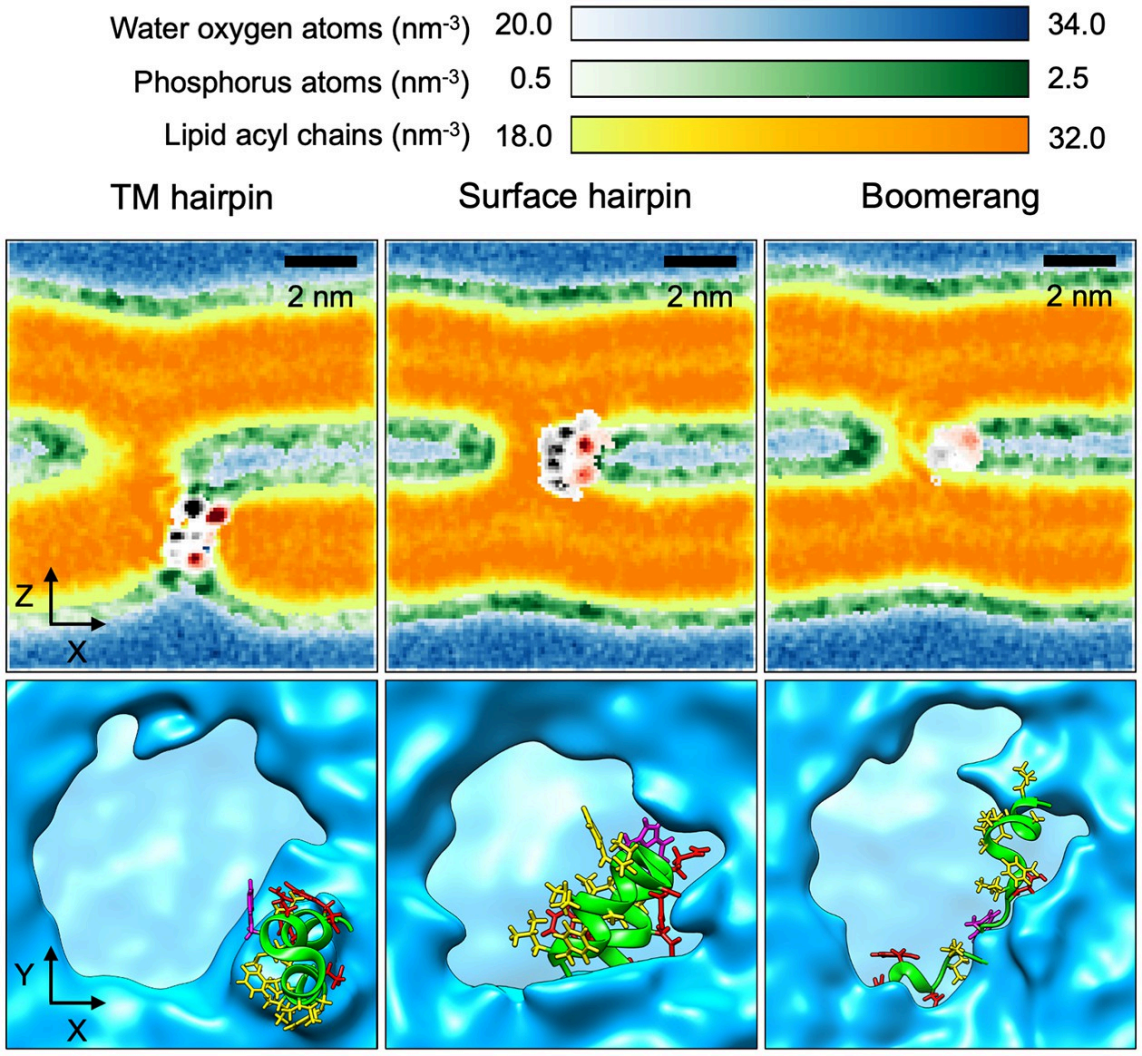
Partial atomic densities across stalk in *XZ* plane (upper row) and simulation snapshots along the *Z* axis (lower row) for TM hairpin, surface hairpin, and boomerang configurations (results for wt boomerang and W14A mutant are qualitatively the same; only the former is shown). Black and red colors denote density of HAfp heavy atoms in hydrophobic and hydrophilic residues, respectively.

The most distinct effect revealed by the PMFs obtained at physiological temperature of 310 K was the stabilisation of stalk structure by the TM wt HAfp. Notably, this system is qualitatively different from all the others, since deeply inserted hairpin induces considerable membrane surface indentation owing to its strong negative hydrophobic mismatch (Fig 6) [34]. As a result, the end-point geometry in this case corresponds to stalk-hole complex [52, 53] rather than to classic stalk structure [10], which is achieved in the remaining scenarios. Favourable energetic contributions in the stalk-hole geometry are expected to arise from the reduction of free energy cost required to maintain highly curved rim around membrane indentation thanks to its relaxation by the neighbouring stalk [53]. Based on coarse grained simulations, such line-tension controlled mechanism was proposed to underlie fusion in the case of SNARE proteins [49], and was also postulated in the context of influenza [43], under the assumption, however, that multiple HAfps in extended, helical configuration are capable of associating into TM bundles. While the existence of such bundles has not been directly confirmed by experiments to date, there is a considerable body of evidence pointing to a tight helical hairpin as the actual fusogenic structure [21, 23, 25, 54]. Although it is nearly twice as short as typical membrane-spanning helices, its ability to maintain metastable intramembrane location was suggested by a number of atomistic simulations to date [33–36, 55]. As evidenced by our recent results, the stability of such deep configuration is further enhanced by *in-vivo* like, trimeric HAfp arrangement, in which intramembrane hairpin units assemble into wedge-like, axially symmetric structures with a central hydrated channel [37].

**Fig 7:**
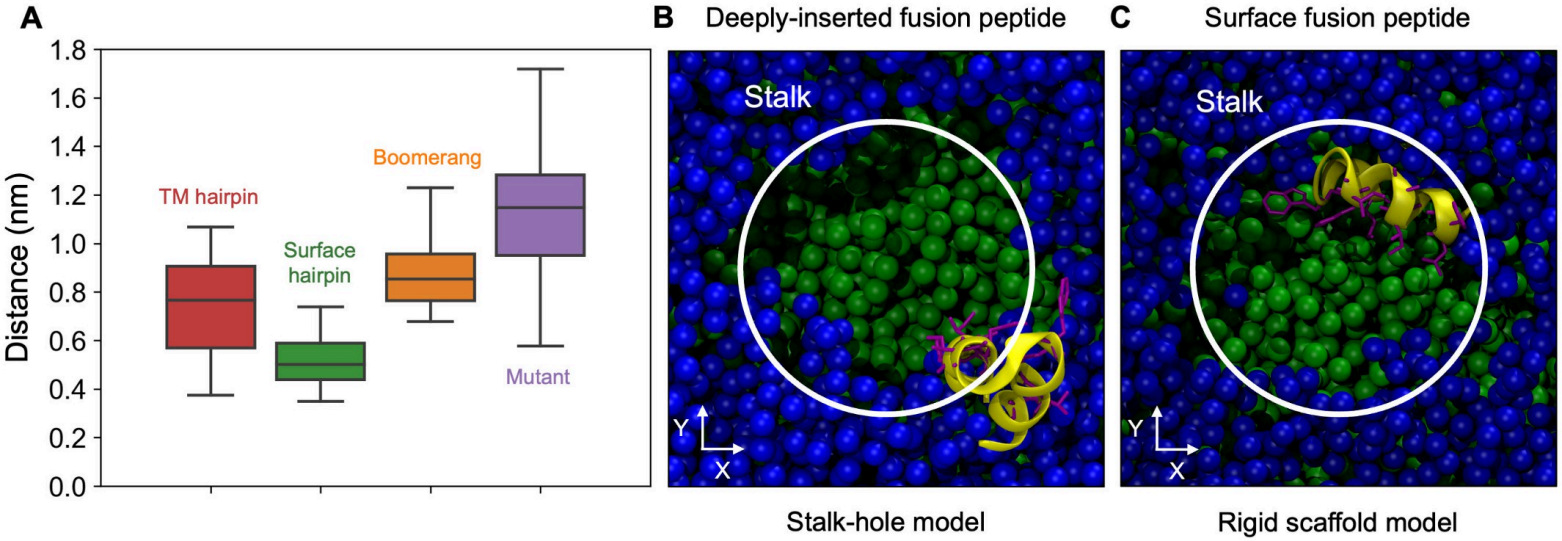
(A) Statistics of distance between C*α* atom of 14th HAfp residue and stalk centre of mass. (B, C) Top view along membrane normal on peptide-stalk assembly summarising the two energetically favourable models. Yellow ribbons—HAfp, green spheres—lipid carbon atoms, blue spheres—water oxygen atoms.

In contrast to the above, none of surface bound HAfp variants appeared to be able to stabilise stalk at 310 K, despite lowering *G*^‡^. For the region of *ξ* > 0.8, free energy profiles obtained in their presence had similar slope to the one for pure membrane system, indicating similar generalised force acting for stalk disassembly. In the case of wt HAfp in hairpin conformation, which produced the lowest *G*^‡^ among surface variants, stalk formation coincides with a sharp change of peptide orientation with respect to membrane plane from roughly membrane parallel, i.e. laying flat, to perpendicular (Fig 5D), in which individual helices interact with opposite membranes. Such a bridging configuration forms a rigid scaffold that supports stalk structure and shields its nonpolar core from water confined within the interbilayer space (Fig 6). Notably, this arrangement is very well compatible with polarity and relative sizes of the two hairpin faces: an extended hydrophobic one, which now spans stalk interior, and a small hydrophilic one, which fits into the narrow aqueous compartment. The above compatibility is also reflected by the tightest and most stable (i.e. with the smallest range of fluctuations) association of the central HAfp C*α* atom from residue 14 with the stalk among all considered surface variants (Fig 7A).

Both wt and W14A mutant HAfp in boomerang configurations were observed to wrap around the stalk, however, in contrast to the stacked helices of the surface bound hairpin structure, they were unable to provide a rigid scaffold that would support its hydrophobic core across the entire extent of the interbilayer space (Fig 6). In addition, their naturally greater conformational variability likely affected the ability to maintain favourable positions of hydrophobic and polar side chains with respect to water and membrane components. This lack of stable arrangement was also evidenced by larger and more fluctuating distance between the C*α* atom of residue 14 and stalk centre than in the case of surface-bound hairpin structure (Fig 7A). Taken together with the highest cost of stalk formation at 350 K among all systems, indicating comparably least favourable entropy contribution, it leads to the conclusion that the presence of boomerang structures likely limits the amount of lipid configurations that are compatible with stalk geometry.

## Conclusion

Our simulations were designed to investigate the effect of HAfp on the formation of initial contact between two, already apposed lipid bilayers. As such they do not account for the work necessary to overcome hydration repulsion that arises when two membranes are brought into close proximity. In this respect, the peptides presence may provide some favourable impact judging by their tendency to promote the dehydration of the interbilayer space observed in unconstrained MD runs. It is unlikely, however, that this effect would constitute their major contribution to the fusion process, since the degree of dehydration caused by wt HAfp was no different than that of fusion-incompetent W14A mutant.

Likewise, our results do not point to any specific effect of HAfp presence on the perturbation of proximal leaflets in the apposed membranes. Within considered simulation time, we did not observe peptides tendency to cross the interbilayer space such that to aid in the formation of initial lipid bridge. The analysis of peptide-induced lipid splays also did not offer plausible explanation for activity, as the frequency of such events was not much higher than in the membrane-only system, and further, was not negatively affected by the W14A mutant. These observations were corroborated by the lack of any qualitative differences in the PMFs at early stages of stalk formation that would relate to peptides presence or their expected fusogenic capacity. This may cast some doubt on the simulation results, however, one should take into account that stalk formation involves free energy barriers in the range of few tens of *k*_*B*_*T*, whereas peptides related effects need not necessarily manifest already within the lower region of this energy scale explored by unconstrained simulations.

This notion seems to be confirmed by our obtained PMFs, which indicate that peptides presence becomes meaningful only at the later stage of stalk formation, closer to the free energy barrier. In this respect, our analysis points to two modes of action, depending on HAfp location within the target membrane. The first one, assuming the existence of membrane-spanning configuration, corresponds to the stalk-hole model (Fig 7B), similar to the one described recently for TM domains of SNARE proteins [49]. It relies on the reduction of local bilayer tension that is achieved by stalk formation in the vicinity of a membrane defect caused by penetrating protein. On macroscopic scale this mechanism can be accompanied by content leakage and, possibly, membrane rupture, if the population of TM hairpins would be high enough. The second mode of action, involves surface bound HAfp hairpin and relies on its ability to support the stalk by adopting orientation perpendicular to membranes plane and forming a rigid scaffold that shields the hydrophobic core from water present in the interbilayer space (Fig 7C). This mechanism corresponds to a classic, non-leaky fusion route. The obtained free energy profiles indicate that the thermodynamic effect of stalk stabilisation in this mechanism is not as strong as in the previous one. This analysis does not include, however, the cost of prior hairpin translocation from surface into the membrane core, which, according to our estimates, may be as high as 40 kJmol^−1^ [36]. Accordingly, the actual preference towards one of the two fusion routes may be modulated by target membrane properties, such as composition or lateral stress, or by cooperative effects of multiple peptide units affecting cis leaflet organisation, making the membrane more or less permissive for TM hairpin. The existence of such alternative scenarios may be in line with two experimentally observed phenotypes of HA-driven fusion. One of them, induced by cholesterol depletion within the target membrane, apparently involves bilayer rupture, and second, favoured in physiological conditions, appears to proceed via classic stalk structure [12]. It is conceivable that low cholesterol content, reducing membrane thickness and rigidity, may indeed increase the population of HAfp in TM configuration, thus promoting the stalk-hole route.

Our results indicate inactivity or at least much lower activity of HAfp in boomerang conformation compared to helical hairpin geometry. The existence of extended structures in TM arrangement does not seem to agree with experimental nor simulation-based evidence gathered to date, making the stalk-hole mechanism less likely in their case. In turn, surface bound configurations appear to be ineffective in stalk stabilisation. Although they were found to lower the enthalpy of stalk containing systems, they appear to also restrict the amount of available configurations consistent with early lipid bridge, thus unfavourably contributing to the entropic component. Notably, the above effects were quantitatively identical in both considered boomerang HAfp versions, of which one contained W14A fusion abrogating mutation. This suggests that the major role of the Trp14 residue in the fusion process may be indirect and related to the stabilisation of hairpin structure [21, 24], and, possibly, facilitation of its transition from surface to TM configuration [36]. The same reasoning may explain high conservation of a number of glycine residues arranged in GxxxG motifs within HAfp sequence [21], since they are necessary for tight packing of the hairpin helices.

## Materials and methods

We considered double membranes systems, containing 23 amino acid long HAfps (wt sequence: GLFGAIAGFIEGGWQGMVDGWYG), representing four configurations, TM hairpin, surface hairpin, boomerang, and fusion-inactive W14A mutant (Fig 2), as well as an analogous membrane only system. The initial hairpin conformation was obtained from NMR structure of lipid-bound HAfp (PDB: 2KXA) [21]. In the case of boomerang and W14A mutant, initial conformations were taken as representative geometries from our earlier peptide-membrane simulations [36]. Lipid bilayers were composed of 308 POPC, 268 POPE, and 308 cholesterol molecules, mimicking an endosomal membrane composition [56]. The system was filled with water molecules described by the TIP3P model [57], and Na^+^Cl^−^ ions in 150 mmol/L concentration. Peptides and lipids were modelled with Amber99SB-ILDNP* [58] and Amber Lipid14 [59] force fields, respectively. Lengths of bonds to hydrogen atoms were fixed using LINCS method [60], and a simulation time step of 2 fs was used. Electrostatic interactions were treated with the particle mesh Ewald method [61], the cutoff for van der Waals interactions was 1 nm, and a long range dispersion correction was used. During production runs, the systems were simulated at temperature 310 K or 350 K maintained by Nosé-Hoover thermostat [62], and pressure of 1 bar maintained with semi-isotropic Parrinello-Rahman barostat [63]. Double-membrane systems were assembled from two separate single bilayer systems constructed with the use of CHARMM-GUI server [64] and initially equilibrated according to its standard protocol. The geometry of double-membrane systems was adjusted such that to provide 3 nm separation between bilayers across periodic system boundary in *Z* direction perpendicular to their planes. The thickness of peptide containing interbilayer region was equilibrated to ∼1 nm by iterative tuning the amount of enclosed water and salt molecules (Fig A in S1 Text). The respective separation of opposing membrane leaflets was measured according to average *Z* positions of their phosphorus atoms in regions localised more than 2 nm away from HAfp heavy atoms. All MD simulations were carried out with Gromacs software [65].

Pairs of unconstrained runs for each considered system were started from the same initially equilibrated geometry, with random velocities. Following short re-equlibration of temperature and pressure, in each case 100 ns were used to provide for departure from the initial structure and subsequent 400 ns (or 500 ns in the case of surface-bound HAfp hairpin) were use for analysis.

All free energy runs were carried out with a modified Gromacs program that implemented a collective variable originally introduced to study pore formation within lipid membranes [66], and subsequently extended to handle stalk nucleation in coarse grained simulations [50, 67]. Briefly, the method considers a cylinder within the interbilayer space that is oriented along the membrane normal and decomposed into slices. The value of collective variable *ξ* ∈ [0, 1], reflects the fraction of slices filled with a predefined number of hydrophobic lipid beads, with *ξ* = 0 corresponding to an empty cylinder (no lipid bridge) and *ξ* = 1 to a fully formed stalk. The entire range of the reaction coordinate can be spanned using umbrella sampling method, with harmonic biasing potentials centred at equally spaced fractional *ξ*_*c*_ values: $V\left(\xi \right)=\frac{1}{2}k{\left(\xi -\xi_{c}\right)}^{2}$. We adapted this methodology to atomistic lipid representation by choosing only specific carbon atoms along acyl chains of POPC and POPE molecules to define the collective variable (Fig B in S1 Text). In our implementation, the cylinder had a radius of 1.2 nm and was partitioned into 18 slices of 0.1 nm thickness. The occupancy factor, *ζ*, which governed the desired level of slice filling was set to 0.5 (see the original work for details [50, 66]).

For each system, starting from two planar bilayers we performed a preliminary slow growth simulation for 100 ns, gradually changing *ξ*_*c*_ from 0.1 to 1 at temperature *T* = 350 K, using *V*(*ξ*) with a force constant *k* = 3000 kJ mol^−1^. Production windows at *ξ*_*c*_ spaced by 0.1, were simulated for at least 300 ns at *T* = 350 K. To determine the necessary amount of sampling in each window, we analysed the convergence of distributions $\rho_{t_{1},t_{2}}\left(\xi \right)$ accumulated within simulation time interval *t*_1_ to *t*_2_. A simulation was extended to time *t*_*e*_, until an interval *t*_*e*_ − *t*_*s*_ ≥ 100 ns, with a midpoint at $t_{h}=\frac{1}{2}\left(t_{s}+t_{e}\right)$, was reached such that the difference between *ρ*(*ξ*) from its first and second halves, $\delta (\rho_{t_{s},t_{h}},\rho_{t_{h},t_{e}})$, was sufficiently small. We used Jensen-Shannon divergence to compare the distributions and adopted a threshold *δ* ≤ 0.1 to terminate simulations. Finally, *ξ* accumulated in [*t*_*s*_, *t*_*e*_] interval were taken for PMF calculations.

Free energy profiles (PMFs) and their uncertainties were computed using the weighted histogram analysis method (WHAM) [68] and bootstrapping technique, respectively, as implemented in Gromacs wham module. In order to obtain PMFs at *T* = 310 K we extended umbrella windows simulated at *T* = 350 K, applying the above convergence validation procedure with the requirement of at least 400 ns simulation time per window at 310 K.

To obtain mean *θ* angle of HAfp hairpin tilt with respect to membrane as a function of the reaction coordinate based on biased umbrella sampling simulations, we used reweighting procedure employing 2-dimensional WHAM calculations [69] as a function of (*ξ*, *θ*), with the force constant for the latter put to 0. The values of mean *θ*(*ξ*) were obtained based on Boltzmann-averaged projection of *θ* distribution on the *ξ* axis.

In our earlier study [36] TM HAfp proved to be metastable with ∼ 5 kJmol^−1^ free energy barrier for escaping to the membrane surface. In order to avoid problems related to uncontrolled HAfp surfacing during PMF calculation, we introduced an additional harmonic restraining potential that enhanced the stability of membrane-spanning configuration in this system. It was defined by an angle between the axis of the N-terminal HAfp helix and the *Z* system axis, with reference value of 0. We evaluated the thermodynamic contribution of this potential in each umbrella window and implemented a necessary correction to the PMF. In addition we verified the stability of TM HAfp in series of unconstrained simulations at *ξ* = 0.2 and *ξ* = 1.0 (for details see Section 3 in S1 Text).

Instantaneous interbilayer distance maps were obtained by calculating membrane separations for a set of {*p*} cartesian grid points in *XY* plane, spaced by 0.5 nm. At a given simulation frames, local interbilayer separation at point *p*(*x*, *y*), was evaluated as the distance along the *Z* axis, *d*_*z*_(*x*, *y*), between maxima of Gaussian smoothed (kernel width 0.6 nm) densities of phosphate atoms, Γ(*x*, *y*, *z*), each from one of the two opposing membrane leaflets: *d*_*z*_(*x*, *y*) = max_*z*_(Γ_1_(*x*, *y*, *z*)) − max_*z*_(Γ_2_(*x*, *y*, *z*)). Final maps were averaged over simulation frames spaced by 1 ns.

Lipid splays were defined by a situation, in which any carbon atom from acyl chains was found further than 0.2 nm from the membrane centre compared to the phosphate atom of the same molecule. Direct interactions between lipids from the opposing leaflets were identified, if the distance between terminal nitrogen and phosphorus atoms of two such lipids were smaller than 0.4 nm. The onset of discontinuity in hydration of the interbilayer space along stalk progression was assessed by evaluating in each umbrella sampling window the average number of intermembrane water oxygen atoms in 625 square bins spanning the *XY* plane and considering the minimum obtained value. For normalisation purpose, the result was divided by the minimal value obtained in membrane only system at *ξ* = 0.1. Spatial density maps for water oxygen atoms, lipid carbon atoms, and lipid phosphate atoms were evaluated by counting the occurrences of respective atom centres in cartesian voxels of 0.1 nm edge. All analyses were performed using MDAnalysis library [70].

## Supporting information

S1 Text**Fig A**. Interbilayer distances in MD simulations for HAfp and no peptide membrane systems in (A) 310 K and (B) 350 K. Error bars correspond to standard deviations of simulation-based ensembles. **Fig B**. Schematic presentation of applied *ξ* potential on fully-atomistic POPC and POPE lipid models. **Fig C**. Left: schematic presentation of correction to the PMF arising due to the presence of restraints in TM HAfp system. $G\left(\xi_{c}\right)=\tilde{G}\left(\xi_{c}\right)+G_{\Phi }\left(0.2\right)-G_{\Phi }\left(\xi_{c}\right)$, where $\tilde{G}\left(\xi_{c}\right)$ is the PMF obtained based on umbrella sampling in restrained TM HAfp system, and *G*(*ξ*_*c*_) is the PMF in which the effect of the restraining potential is removed. Right: *G*_Φ_(*ξ*_*c*_) values obtained for all umbrella sampling windows. **Fig D**. Values of *θ* angle obtained in unrestrained simulations of TM HAfp at *ξ*_*c*_ = 0.2 and *ξ*_*c*_ = 1.0. **Fig E**. Interbilayer separation in membrane only system in two independent simulations (upper and lower row, respectively) presented for 4 × 100 ns simulation blocks. **Fig F**. Interbilayer separation in membrane only system averaged over combined 400 ns from two independent simulations. **Fig G**. Thickness of interbilayer space averaged over 2 × 400 ns from independent MD runs. Positions of HAfp C*α* atoms every 100 ns are marked with circles; blue: residues 1, 7, 9, green: residue 14, red: residues 17, 21, 23; positions of N and C termini are marked by blue and red stars, respectively. Black dots correspond to locations of N-P contacts. **Fig H**. Ratios of lipid tails protrusions per unit simulation time within HAfp-containing membrane in unconstrained simulations (free MD) and subsequent umbrella sampling windows (denoted by *ξ*_*c*_) to protrusions in unconstrained membrane-only system. **Table A**. Comparison of *G*_Φ_ values for *ξ*_*c*_ = 0.2 and *ξ*_*c*_ = 1.0, obtained based on restrained ($G_{\Phi }^{+}=+k_{B}T\;\text{ln}{\langle \text{exp}\left(\beta \Phi \right)\rangle }_{\xi_{c}}^{\Phi }$), and unrestrained ($G_{\Phi }^{-}=-k_{B}T\;\text{ln}{\langle \text{exp}\left(-\beta \Phi \right)\rangle }_{\xi_{c}}$) MD runs. **Table B**. List of unconstrained (i.e. without biasing umbrella potential) MD runs for pre-stalk systems. **Table C**. Summary of umbrella sampling simulations at *T* = 350 K. A total simulation time and final simulation interval used for PMF calculation (in parenthesis) are given for each window. *δ*—the achieved level of convergence, estimated as the Jensen-Shannon divergence between *ξ* distributions obtained for the first and the second halves of the final interval used for PMF calculation. **Table D**. Summary of umbrella sampling simulations at *T* = 310 K. A total simulation time and final simulation interval used for PMF calculation (in parenthesis) are given for each window. *δ*—the achieved level of convergence, estimated as the Jensen-Shannon divergence between *ξ* distributions obtained for the first and the second halves of the final interval used for PMF calculation. **Table E**. Comparison of basic membrane parameters obtained with the force field and simulation setup used in the current study and experiment, based on 1 *μs* simulation of planar membrane composed of 100 POPC lipids at 310 K.(PDF)
