# Supplementary material for: Two modes of fusogenic action for influenza virus fusion peptide
Source: PLoS Comput Biol. 2023 May 26;19(5):e1011174. doi: 10.1371/journal.pcbi.1011174 (PMC10249882; doi:10.1371/journal.pcbi.1011174)
Supplement: S1 Text — Fig A. Interbilayer distances in MD simulations for HAfp and no peptide membrane systems in (A) 310 K and (B) 350 K. Error bars correspond to standard deviations of simulation-based ensembles. Fig B. Schematic presentation of applied ξ potential on fully-atomistic POPC and POPE lipid models. Fig C. Left: schematic presentation of correction to the PMF arising due to the presence of restraints in TM HAfp system. G(ξc)=G˜(ξc)+GΦ(0.2)-GΦ(ξc), where G˜(ξc) is the PMF obtained based on umbrella sampling in restrained TM HAfp system, and G(ξc) is the PMF in which the effect of the restraining potential is removed. Right: GΦ(ξc) values obtained for all umbrella sampling windows. Fig D. Values of θ angle obtained in unrestrained simulations of TM HAfp at ξc = 0.2 and ξc = 1.0. Fig E. Interbilayer separation in membrane only system in two independent simulations (upper and lower row, respectively) presented for 4 × 100 ns simulation blocks. Fig F. Interbilayer separation in membrane only system averaged over combined 400 ns from two independent simulations. Fig G. Thickness of interbilayer space averaged over 2 × 400 ns from independent MD runs. Positions of HAfp Cα atoms every 100 ns are marked with circles; blue: residues 1, 7, 9, green: residue 14, red: residues 17, 21, 23; positions of N and C termini are marked by blue and red stars, respectively. Black dots correspond to locations of N-P contacts. Fig H. Ratios of lipid tails protrusions per unit simulation time within HAfp-containing membrane in unconstrained simulations (free MD) and subsequent umbrella sampling windows (denoted by ξc) to protrusions in unconstrained membrane-only system. Table A. Comparison of GΦ values for ξc = 0.2 and ξc = 1.0, obtained based on restrained (GΦ+=+kBTln〈exp(βΦ)〉ξcΦ), and unrestrained (GΦ-=-kBTln〈exp(-βΦ)〉ξc) MD runs. Table B. List of unconstrained (i.e. without biasing umbrella potential) MD runs for pre-stalk systems. Table C. Summary of umbrella sampling simulations at T = 350 K. [file pcbi.1011174.s001.pdf]

Electronic Supporting Information for:

Two modes of fusogenic action for influenza virus fusion peptide

Michal Michalski[a], Piotr Setny[a,1]

[a] Centre of New Technologies, University of Warsaw, Warsaw, Poland

[1] corresponding author: p.setny@cent.uw.edu.pl

1. Interbilayer separation in considered systems.

Hydration of interbilayer space was tuned to achieve an average separation between two opposing leaflets close $\sim$ 1.0 nm. The separation was measured as a distance along the axis perpendicular to membranes plane (Z axis) between mass centres of respective sets of phosphate atoms. In the case of systems containing hemagglutinin fusion peptides (HAfps), only phosphate atoms that were at least 2 nm away in XY plane from any peptide heavy atom were considered. Average distances obtained in unconstrained molecular dynamics (MD) runs are presented in Fig A.

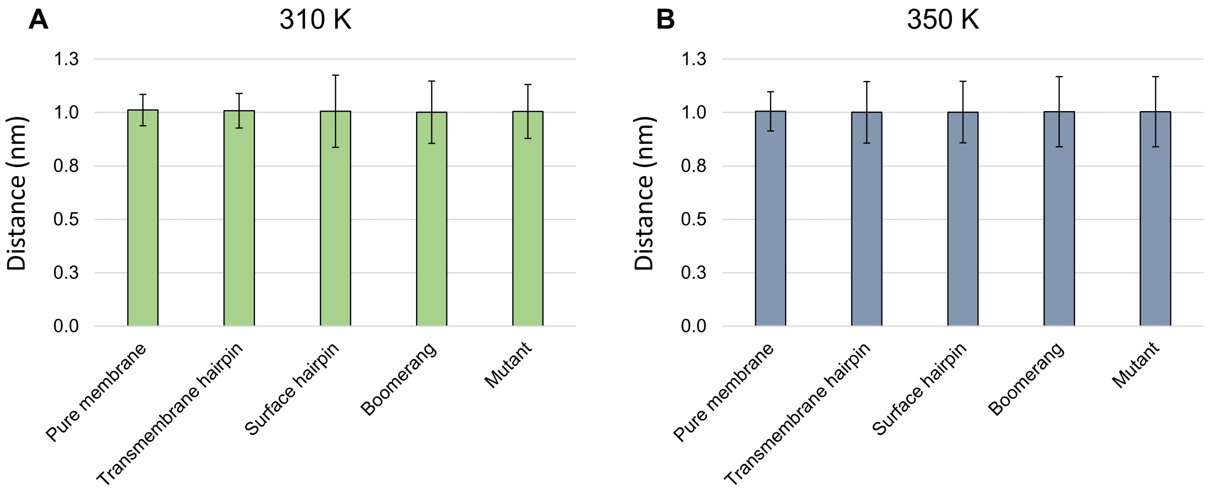

Fig A. Interbilayer distances in MD simulations for HAfp and no peptide membrane systems in (A) 310 K and (B) 350 K. Error bars correspond to standard deviations of simulation-based ensembles.

2. Definition of collective variable

The original implementation of collective variable used to control the progress of stalk formation was based on coarse grained lipid representation [1]. In order to use it in our atomistic simulations we based the definition of collective variable on a subset of carbon atoms within lipid acyl chains (Fig B) that best matched the locations of coarse beads in the original implementation.

Carbon atom	POPC	POPE
1	C115	C115
2	C111	C111
3	C17	C17
4	C13	C13
5	C23	C23
6	C27	C27
7	C210	C210
8	C213	C213
9	C217	C217

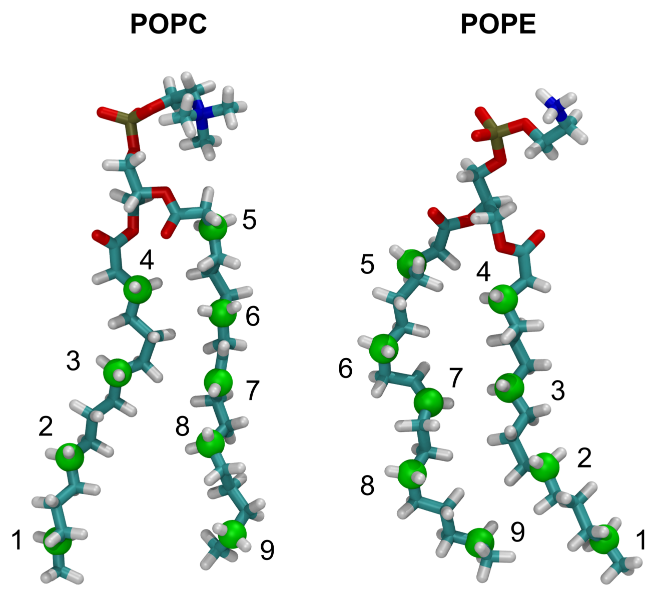

Fig B. Schematic presentation of applied ξ potential on fully-atomistic POPC and POPE lipid models.

3. Harmonic restraints in TM HAfp system

Hairpin structure in the transmembrane (TM) HAfp system was subjected to an additional harmonic restraining potential, $\Phi(\theta) = \frac{1}{2}k_\phi \theta^2$, in order to secure its stability in membrane-spanning configuration during all windows of umbrella sampling simulations. The potential was defined by an angle, θ, between the vector connecting centres of mass of HAfp residues 7-12 and 1-6 (i.e. parallel to the axis of the N-terminal helix) and the Z system axis, with a force constant of $k_\phi = 3000$ kJ mol^{-1}rad^{-2}.

The influence of the restraining potential on the potential of mean force (PMF) in the TM HAfp system was taken into account by introducing a correction $\Delta G_\Phi(\xi_c) = G_\Phi(0.2) - G_\Phi(\xi_c)$, that represented free energy difference between the introduction of restraints at $\xi_c = 0.2$, and their removal at a given ξ_c (Fig C, left). The value of $G_\Phi(\xi_c)$ was estimated using the free energy perturbation formula: $G_\Phi(\xi_c) = +k_B T \ln\langle \exp(\beta\Phi)\rangle^{\Phi}_{\xi_c}$, where $k_B T$ is the Boltzmann constant times temperature, $\beta = (k_B T)^{-1}$, and $\langle . \rangle^{\Phi}_{\xi_c}$ denotes an average obtained at umbrella window centred at ξ_c, and simulated in the presence of the restraining potential. $G_\Phi(\xi_c)$ values obtained for all umbrella sampling windows are shown in Fig C, right.

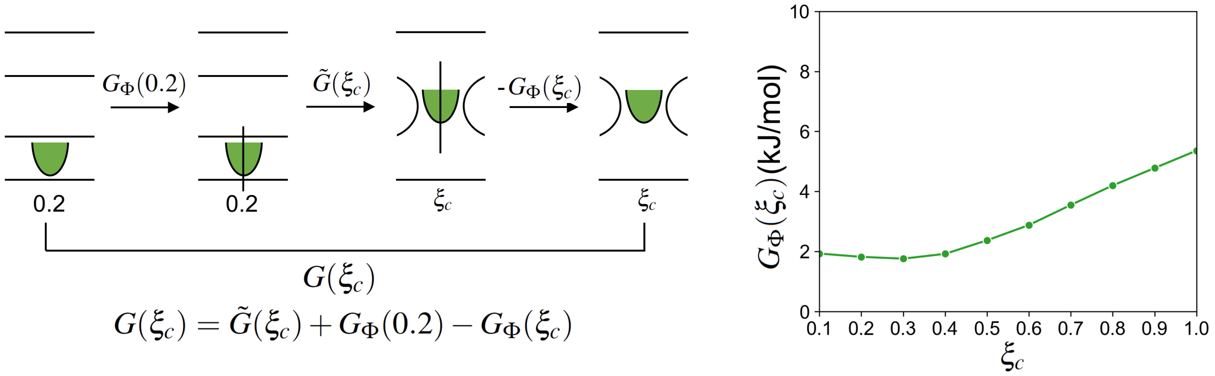

Fig C. Left: schematic presentation of correction to the PMF arising due to the presence of restraints in TM HAfp system. $G(\xi_c) = \tilde{G}(\xi_c) + G_\Phi(0.2) - G_\Phi(\xi_c)$, where $\tilde{G}(\xi_c)$ is the PMF obtained based on umbrella sampling in restrained TM HAfp system, and $G(\xi_c)$ is the PMF in which the effect of the restraining potential is removed. Right: $G_\Phi(\xi_c)$ values obtained for all umbrella sampling windows.

In order to prove the intrinsic stability of TM hairpin, we carried out 3 sets of MD runs per 300 ns each of TM HAfp system with no restraining potential at umbrella sampling windows corresponding to $\xi_c = 0.2$ and $\xi_c = 1.0$. The statistics of θ values from the last 100 ns from each run (Fig D) indicate that HAfp is indeed capable of maintaining membrane-spanning configuration both prior to and after stalk formation.

In addition, the above runs served for the estimation of free energy cost for introducing the restraints based on alternative form of free energy perturbation formula: $G_\Phi(\xi_c) = -k_B T \ln\langle \exp(-\beta\Phi)\rangle_{\xi_c}$, in which the averaging is performed over unconstrained states. These calculations allowed the assessment of hysteresis error in $G_\Phi(\xi_c)$ calculations. Based on the obtained values (Table 1) we estimate this error to be in the range of 1 kJmol^{-1}, i.e. well below the overall PMF uncertainty.

Table A. Comparison of G_Φ values for $\xi_c = 0.2$ and $\xi_c = 1.0$, obtained based on restrained ($G^+_\Phi = +k_B T \ln\langle \exp(\beta\Phi)\rangle^{\Phi}_{\xi_c}$), and unrestrained ($G^-_\Phi = -k_B T \ln\langle \exp(-\beta\Phi)\rangle_{\xi_c}$) MD runs.

ξ_c	G^+_Φ (kJmol^{-1})	G^-_Φ (kJmol^{-1})
0.2	1.86	1.81
1.0	5.36	6.64

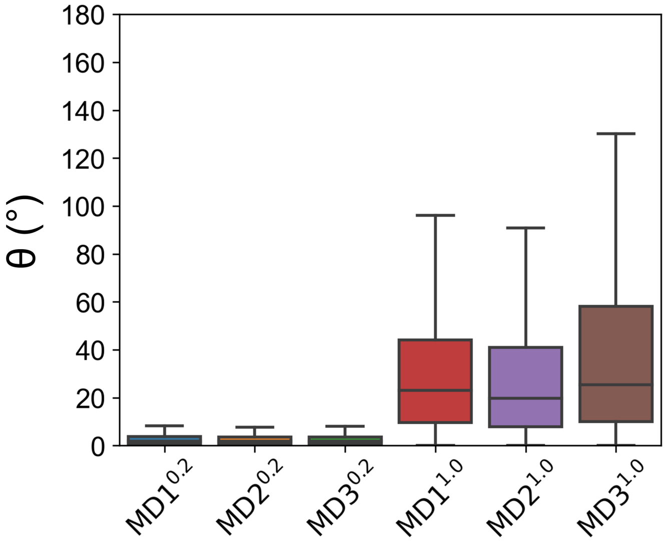

Fig D. Values of θ angle obtained in unrestrained simulations of TM HAfp at $\xi_c = 0.2$ and $\xi_c = 1.0$.

4. Fluctuations of interbilayer distance in membrane only system.

A peptide-free system of two bilayers separated by 1 nm of aqueous solvent was simulated in analogous conditions to systems with HAfps to provide insights into the amplitude and timescale of spontaneous fluctuations of local interbilayer distance.

The analysis of interbilayer separation averaged over consecutive 100 ns intervals (Fig E) indicates significant amplitude of distance fluctuations as well as they slow decay.

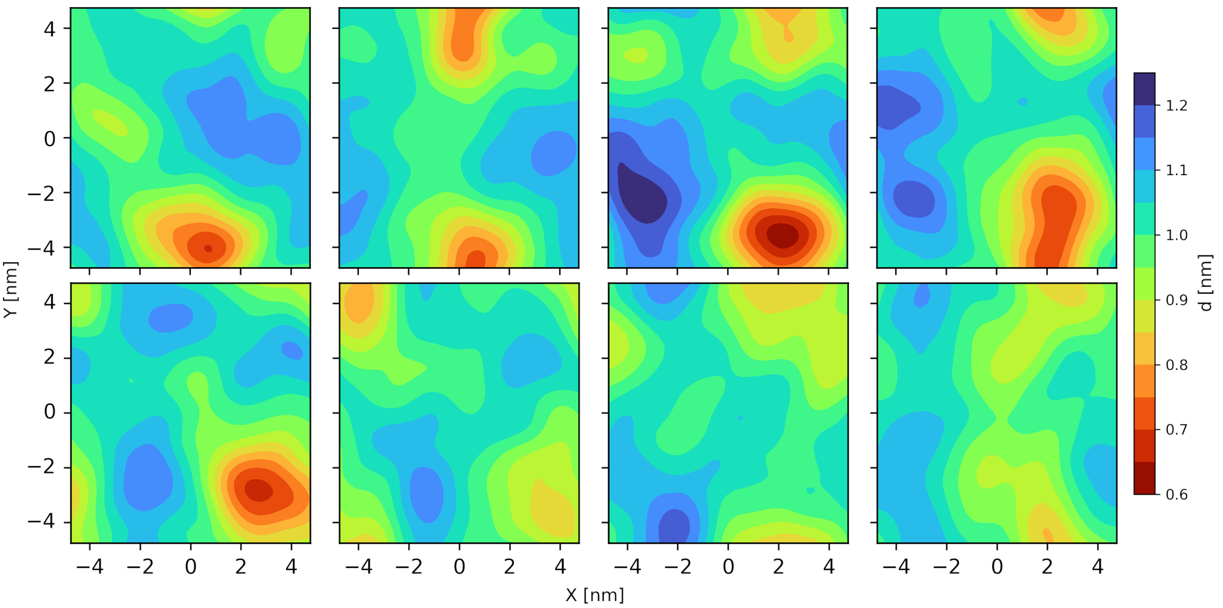

Fig E. Interbilayer separation in membrane only system in two independent simulations (upper and lower row, respectively) presented for 4 × 100 ns simulation blocks.

A map of average interbilayer separation based on combined final 400 ns of two independent simulations of membrane only system (Fig F) still indicates deviations from the mean value (1 nm) in the order of ±0.15 nm. The degree of maximum local thinning is smaller than in the systems containing HAfp in boomerang or TM configurations, and similar to the one observed in the case of surface bound HAfp hairpin.

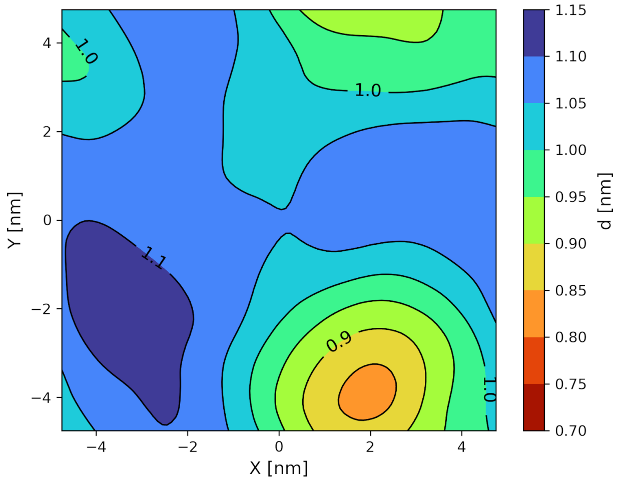

Fig F. Interbilayer separation in membrane only system averaged over combined 400 ns from two independent simulations.

5. Direct contacts between lipids from opposing membranes

The degree of local dehydration was found to positively correlate with the frequency of direct contact (less than 0.4 nm distance between heavy atoms) between polar heads (NP-contacts) of lipids from the opposing membrane leaflets. A spatial map in XY plane showing the distribution of the observed contacts is shown in Fig G.

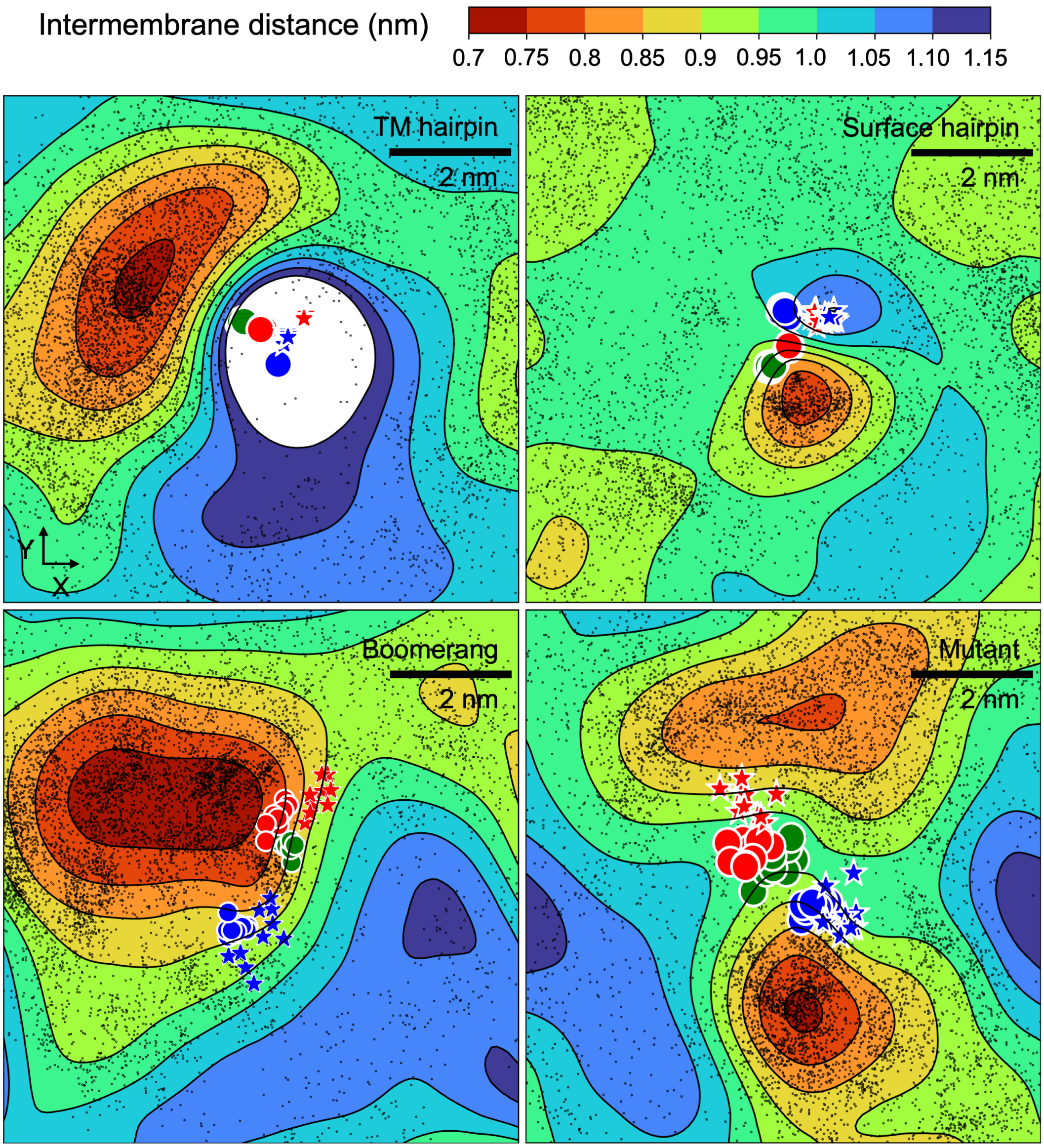

Fig G. Thickness of interbilayer space averaged over 2×400 ns from independent MD runs. Positions of HAfp Cα atoms every 100 ns are marked with circles; blue: residues 1, 7, 9, green: residue 14, red: residues 17, 21, 23; positions of N and C termini are marked by blue and red stars, respectively. Black dots correspond to locations of N-P contacts.

6. Lipid tails protrusions during stalk formation

The frequency of lipid tails protrusion in the target bilayer was monitored during umbrella sampling simulations. The results indicate similar behaviour in all systems containing surface-bound HAfp, including the inactive W14A mutant. Of note is the effect of slight protrusions suppression by the biasing potential used in umbrella sampling.

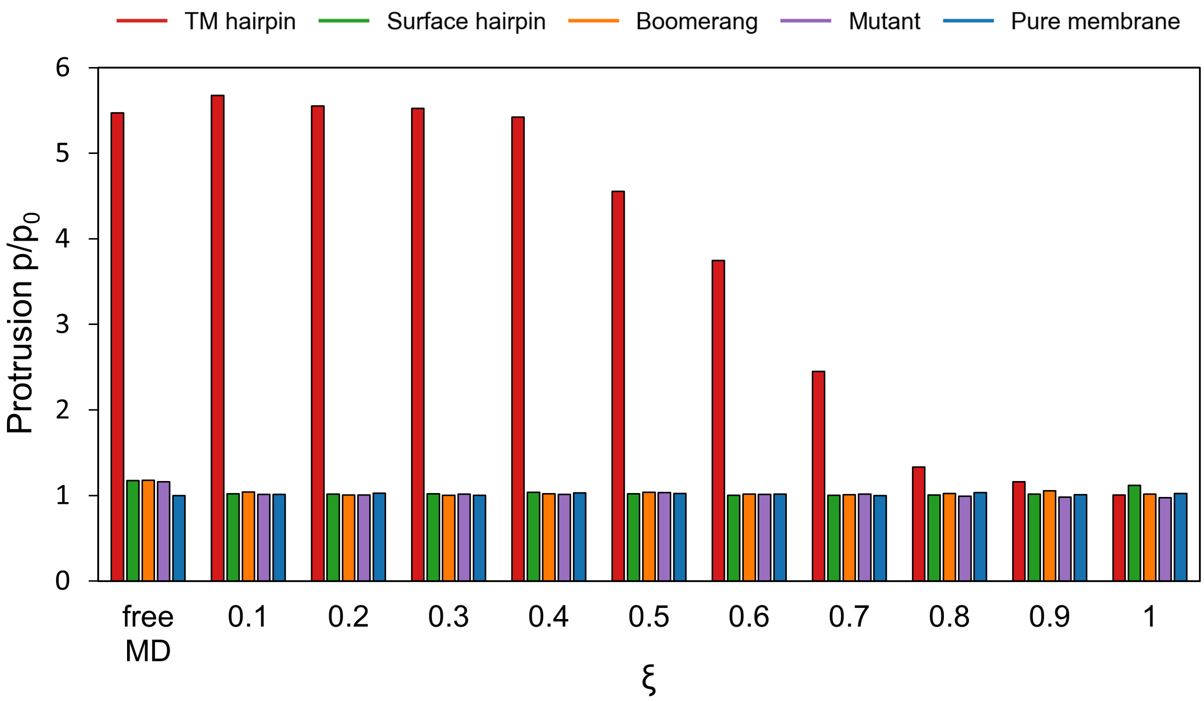

Fig H. Ratios of lipid tails protrusions per unit simulation time within HAfp-containing membrane in unconstrained simulations (free MD) and subsequent umbrella sampling windows (denoted by ξ_c) to protrusions in unconstrained membrane-only system.

7. List of MD simulations

Table B. List of unconstrained (i.e. without biasing umbrella potential) MD runs for pre-stalk systems. The first 100 ns of each run was discarded for analysis.

system	MD1 run (ns)	MD2 run (ns)
HAfp:		
TM	500	500
surface hairpin	600	600
surface boomerang	500	500
W14A mutant	500	500
membrane only	500	500

Table C. Summary of umbrella sampling simulations at $T = 350$ K. A total simulation time and final simulation interval used for PMF calculation (in parenthesis) are given for each window. δ – the achieved level of convergence, estimated as the Jensen-Shannon divergence between ξ distributions obtained for the first and the second halves of the final interval used for PMF calculation.

ξ_c	TM hairpin		Surface hairpin		Boomerang		Mutant		Pure membrane	
	Time (ns)	δ	Time (ns)	δ	Time (ns)	δ	Time (ns)	δ	Time (ns)	δ
0.1	300 (281)	0.06	300 (242)	0.05	300 (282)	0.05	300 (288)	0.05	300 (252)	0.05
0.2	300 (289)	0.07	300 (214)	0.06	300 (237)	0.06	300 (253)	0.06	300 (289)	0.05
0.3	300 (252)	0.07	300 (254)	0.06	300 (285)	0.06	300 (290)	0.07	300 (277)	0.05
0.4	300 (196)	0.05	300 (220)	0.06	300 (290)	0.05	300 (272)	0.05	300 (286)	0.06
0.5	300 (128)	0.08	300 (187)	0.06	300 (282)	0.05	300 (149)	0.06	300 (180)	0.05
0.6	300 (162)	0.07	300 (155)	0.06	300 (255)	0.06	300 (270)	0.05	300 (156)	0.06
0.7	300 (153)	0.09	300 (107)	0.06	300 (159)	0.08	300 (260)	0.06	300 (281)	0.04
0.8	300 (290)	0.07	300 (187)	0.05	300 (289)	0.06	300 (280)	0.05	300 (217)	0.05
0.9	300 (152)	0.05	300 (272)	0.05	300 (290)	0.07	300 (208)	0.05	300 (290)	0.04
1.0	300 (260)	0.03	300 (129)	0.04	300 (256)	0.03	300 (128)	0.06	300 (141)	0.05

Table D. Summary of umbrella sampling simulations at $T = 310$ K. A total simulation time and final simulation interval used for PMF calculation (in parenthesis) are given for each window. δ – the achieved level of convergence, estimated as the Jensen-Shannon divergence between ξ distributions obtained for the first and the second halves of the final interval used for PMF calculation.

ξ_c	TM hairpin		Surface hairpin		Boomerang		Mutant		Pure membrane	
	Time (ns)	δ	Time (ns)	δ	Time (ns)	δ	Time (ns)	δ	Time (ns)	δ
0.1	400 (178)	0.05	400 (390)	0.04	400 (349)	0.04	400 (297)	0.05	400 (271)	0.05
0.2	400 (181)	0.07	400 (299)	0.05	400 (181)	0.09	400 (386)	0.05	400 (384)	0.04
0.3	400 (262)	0.05	400 (270)	0.05	400 (234)	0.08	400 (358)	0.05	400 (275)	0.07
0.4	400 (146)	0.06	400 (389)	0.05	400 (362)	0.05	400 (386)	0.05	400 (229)	0.05
0.5	400 (133)	0.06	400 (389)	0.05	400 (354)	0.04	400 (383)	0.04	400 (275)	0.04
0.6	400 (285)	0.04	400 (320)	0.07	400 (256)	0.05	400 (195)	0.06	400 (299)	0.04
0.7	400 (252)	0.05	400 (209)	0.05	400 (347)	0.03	400 (154)	0.05	400 (180)	0.06
0.8	400 (258)	0.03	400 (390)	0.05	400 (387)	0.04	600 (590)	0.04	400 (246)	0.06
0.9	400 (267)	0.03	700 (315)	0.07	400 (357)	0.04	600 (496)	0.03	400 (272)	0.04
1.0	400 (176)	0.05	400 (387)	0.03	400 (174)	0.02	400 (292)	0.03	400 (159)	0.08

Table E. Comparison of basic membrane parameters obtained with the force field and simulation setup used in the current study and experiment, based on 1 μs simulation of planar membrane composed of 100 POPC lipids at 310 K.

Force Field	Thickness (nm)	APL (nm^2)
Amber Lipid14 [2]	3.78 ± 0.016	0.667 ± 0.012
Experiment	3.93 (300 K) [3]<br>3.82 (320 K) [3]	0.66 (310 K) [4]

References

[1] Jochen S. Hub and Neha Awasthi. Probing a Continuous Polar Defect: A Reaction Coordinate for Pore Formation in Lipid Membranes. *J. Chem. Theory Comput.*, 13(5):2352–2366, 2017.

[2] Callum J. Dickson, Benjamin D. Madej, Age A. Skjevik, Robin M. Betz, Knut Teigen, Ian R. Gould, and Ross C. Walker. Lipid14: The amber lipid force field. *Journal of Chemical Theory and Computation*, 10(2):865–879, 2014. PMID: 24803855.

[3] Norbert Kučerka, Mu-Ping Nieh, and John Katsaras. Fluid phase lipid areas and bilayer thicknesses of commonly used phosphatidylcholines as a function of temperature. *Biochimica et Biophysica Acta (BBA) - Biomembranes*, 1808(11):2761–2771, 2011.

[4] Paul A. Hyslop, Benoit Morel, and Richard D. Sauerheber. Organization and interaction of cholesterol and phosphatidylcholine in model bilayer membranes. *Biochemistry*, 29(4):1025–1038, 1990. PMID: 2160270.
